# Thanksgiving Day

**Published:** 1875-11

**Authors:** 


### THANKSGIVING DAY.

We have none too many holidays for our good, but we greatly need to learn how to use them wisely. The anniversary of the National Independence is a day of license and misconduct, and even our religious holidays are not thoughtfully celebrated. Scotland is about to abolish her fast-days because of the dissipation which they have induced. From time immemorial, Fast-Day in that country has been an occasion for hard drinking, which means the consumption of an enormous amount of the strong, creosote-flavored whisky of that land. When the "day of fasting and prayer" was originally instituted, the intoxication of the day was preceded by attendance at the kirk—the place of worship; but now only a few visit the church at all. The day begins and ends as a day of wild debauch and horrid revelry.

With multitudes of our own people the fact is the same. There is scarcely a thought in the majority of minds concerning the intent and object of the day. Through some formality which they care not to investigate, a holiday is secured, and therefore "a good time" is to be enjoyed. A good time means more or less of undue license; more or less of the unusual gratification of the appetites and passions; more or less of intoxication; and a good deal of headache and misery the next day.

We fear all this will be true of the annual holiday which falls in this month of November. The times are hard, and money and labor and wages are not abundant; but the cost of enough of intoxication to bring a large installment of misery to hundreds of thousands, will be forthcoming somehow. In view of the excesses into which weak humanity is led on such occasions, perhaps it were better to abandon this class of holidays, and strive to make mankind duly thankful on all the days of the year. For the annual "Fast-Day"—which all know is so only in name—we would do well to substitute absence from gluttony all the year round. For the "Glorious Fourth," we would, if wise, seek to give voice to a genuine patriotism at all times. If we think we have anything to be thankful for, let us see if we can not pass the holiday of thanks in a true and worthy spirit, by taking a brighter view of life, whatever may be its hardships; and acknowledging our gratitude for the mercy which is infinite, the charity which is boundless, the gifts which are unceasing without reference to our thoughtlessness and irreverence.

---

### OUR SUMMER AT MAPLEWOOD. CONCLUSION.

Next month the very interesting and valuable story which has run through the pages of the JOURNAL since February, will reach its conclusion. We are justified in saying that the attempt to embody in popular form valuable methods of preparing foods, has not been excelled in modern literature. The recipes given have all been carefully tested, and if scrupulously followed, will never disappoint the housekeeper. We have copyrighted the story, and propose to publish it in an attractive volume, as soon as trade brightens.

---

Blind fortune treads on the steps of inconsiderate rashness.

